# Effect Size as the Essential Statistic in Developing Methods for mTBI Diagnosis

**DOI:** 10.3389/fneur.2015.00126

**Published:** 2015-06-18

**Authors:** Douglas Brandt Gibson

**Affiliations:** ^1^Programs, Budget and Strategies Office, U.S. Army Research Institute, Fort Belvoir, VA, USA

**Keywords:** traumatic brain injury, classical measurement theory, effect size, MACE

## Abstract

The descriptive statistic known as “effect size” measures the distinguishability of two sets of data. Distingishability is at the core of diagnosis. This article is intended to point out the importance of effect size in the development of effective diagnostics for mild traumatic brain injury and to point out the applicability of the effect size statistic in comparing diagnostic efficiency across the main proposed TBI diagnostic methods: psychological, physiological, biochemical, and radiologic. Comparing diagnostic approaches is difficult because different researcher in different fields have different approaches to measuring efficacy. Converting diverse measures to effect sizes, as is done in meta-analysis, is a relatively easy way to make studies comparable.

Mild traumatic brain injury (mTBI) is a recognized clinical entity, but surrounded by diagnostic controversy. It can be safely stated that there is no single operational definition of what constitutes mTBI. An operational definition in terms of a measurement method is needed in order to make the entity of mTBI amenable to scientific investigation. Without a way to measure mTBI, it is difficult to triage, treat, or to develop therapies for it. In this article, I hope to point out some features of an operationalized mTBI diagnostic need to be appreciated in evaluating effectively discriminating diagnostic methods using classical measurement theory.

The idea of an “operational” definition is that instead of positing an abstract concept and trying to come up with a measurement method, one accepts the measurement instrument itself as the definition of the concept. Since we struggle to come up with a definition of mTBI, it should suffice to develop an instrument that can reliably distinguish those who have been concussed from those that have not. The focus should be on distinguishing affected from unaffected individuals or impaired from unimpaired, not what constitutes the abstract term “mTBI.”

The goal of an efficient diagnostic instrument development process is to maximize the discriminability between an impaired population and an unimpaired (in other words, normal or control) population, in this case, between those impaired by concussion and those who are unimpaired. “Effect size” is a measure of this discriminability. It can be used to choose between alternative methods of diagnosis and, in development, to gage improvements in a diagnostic method. For comprehensive reviews of the effect size statistic, see Kelley and Preacher (1), and McGough and Faraone (2).

## Effect Size

There is more than one definition of effect size. For the sake of clarity of exposition this article uses Cohen’s *d* (3). Correlation is often cited as an effect size measure but one must be clear about the type of correlation being used. Methods that measure the pair-wise association of values on different scales do not directly correspond to the measures of discrimination between collections of values on a single scale that are addressed here. In short, calculation of Cohen’s *d*, is independent of calculation of Pearson’s *r*. Breaking up the pairing of values drastically changes the correlation, but the effect size as used above does not change. Glass’s delta is interesting in that it does not used the pooled SD as Cohen’s *d* does, but uses the SD of the control group. On the one hand, a well-standardized control group might provide very good reference values against which to test other populations. Pathological populations are generally more variable, so this might be a good approach. On the other hand, using the variance of only one population distorts the picture and does not give the empirically true difference. This is the basis of the controversy over using *p* values, that is, type I errors are controlled, but type II errors are not. Other measures, extensions of the effect size concept, are available for multivariate use, categorical variables, and to correct for bias. It is said that there are over one hundred effect size measures.

**Figure 1:**
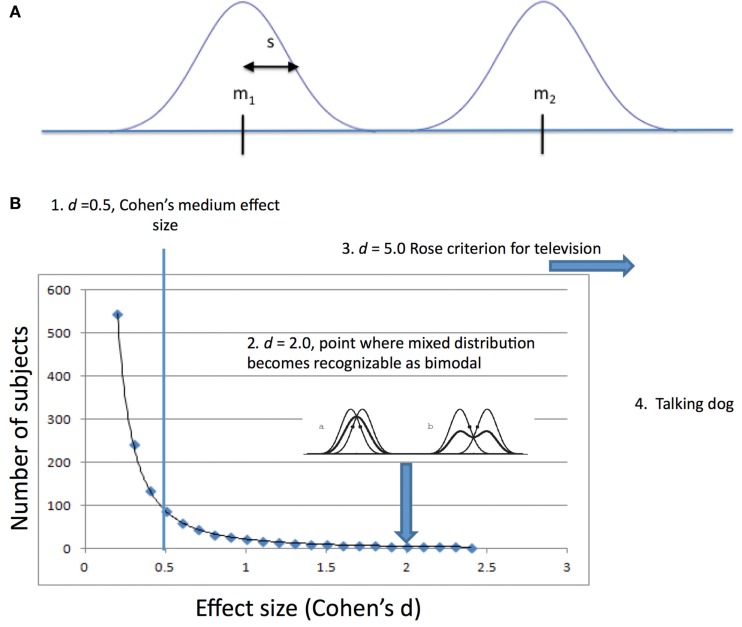
**(A)** Variables involved in measuring discriminability using Cohen’s *d*. *m*_1_ is the mean of one population, *m*_2_ is the mean of the other population, and *s* is a measure of the dispersion of individual values around the mean, for Cohen, the “pooled” standard deviation. **(B)** 1. Cohen’s small, medium, and large values for effect size were given in the context of psychological research where, because psychological differences measurable between people are small, large numbers of subjects are needed to reveal them. 2. A mixture of two distributions of the same variance must be two (2.0) SDs apart before the Gaussian curve becomes bimodal (second derivative becomes concave down). 3. Rose’s criterion for discriminability of signals in television requires *d* of 5.0. 4. If you have a talking dog, you just need one to prove your point. Perhaps two if you insist on a control dog.

Effect size measures take two factors into account, the difference between the mean values of the measures for the two groups and the variance (roughly the spread of the data points for each group; more exactly, the squared SD of the sample). Here is a simple way of stating effect size:

$$d=\left(m_{\text{2}}-m_{\text{2}}\right)∕\text{s}$$

where *m*_1_ is the mean of one population, *m*_2_ is the mean of the other population, and *s* is the “pooled” SD. The effect size, *d*, is often referred to as Cohen’s *d* (Figure 1A).

There is some debate about the SD, *s*, in cases where the two populations have different SDs. One school of thought advises using the SD of the control population (in this case, the unimpaired population). For mTBI studies, the impaired population is more heterogeneous than the unimpaired population because of, for instance, differences in time elapsed since exposure and different levels of severity. Cohen “pooled” the SDs, in effect comparing a larger-than-measured SD for the unimpaired population and a smaller-than-measured SD for the impaired population.

The difference between the means can be larger or smaller than the SD. Therefore, *d* can vary from close to 0 to infinity. Where the difference between the means is equal to the SD, is 1. When the difference between the means is less than the SD, *d* is between 0 and 1. When the SD is less than the difference between the means, *d* is >1.

Effect size is of interest in mTBI diagnosis because it allows us to compare diagnostic measures across methodology, whether biochemical, electrophysiological, radiological, or behavioral.

Especially of interest is that psychological measures generally have effect sizes with *d* <1. Cohen in 1991 estimated that for psychological tests *d* = 0.80 should be considered a large effect size. Table 1 listed the *d* levels that Cohen considered large, medium, and small along with the proportion of time a diagnosis based on those effect sizes would be correct. As the table shows a diagnosis based on a test with a large Cohen’s *d* would only be correct only 71% of the time.

**Table 1:** **Cohen suggested that, for studies in psychology, effect sizes be described as “small,” “medium,” or “large”**.

Cohen	*d*	% Correct
Large	0.8	71
Medium	0.5	64
Small	0.2	56

*The corresponding effect size values of d are given here along with the corresponding “areas under the receiver operating characteristic (ROC) curve” or AUC, here given as “% Correct,” or the percentage of correct determinations one would make (both true positives and true negatives) if a test of that effect size were used to make a single decision*.

A psychological test with an effect size as great as 1 would be considered exceptionally high. In other fields, effect sizes as low as 1 are the exception. In communication engineering, a minimum effect size is 5. This is the so-called “Rose criterion” (4). For some observations, the phenomenon is so obvious and effect size is so great that statistical tests of the distinguishability become irrelevant. This latter type of observation is pathognomonic of the condition.

Consider the clinician who bases an individual diagnosis of mTBI on a test with a small effect size. Suppose the effect size is 0.2. What percentage of such diagnoses would be correct? From the chart above, we can see that only 56% of the clinician’s diagnoses would be correct, hardly better than chance.

On the other hand, consider an epidemiologist who uses a small effect size to recommend a general triage rule for triage of patients with mTBI. If the rule were applied to 100 patients, the rule would result in 6 fewer misdiagnoses (false negatives as well as false positives) when compared to random assignment.

Psychological tests are often the go-to choice for mTBI diagnosis. But, an effect size of <1 means that the mistakes in diagnosis of individuals will probably be unacceptably high. The diagnostician will encounter both a high-false positive rate and a high-false negative rate. Patients with the disorder will be falsely deemed unimpaired and patients without the disorder will be falsely deemed impaired.

TBI diagnostics fall into four broad groups: psychological, physiological, radiological, and biochemical. Psychological tests are generally inventories of symptoms either observed or patient-reported. Physiological tests include tests of balance, eye-movements, EEG, and so on. Radiological tests are based on imaging and include such promising approaches a diffusion tensor imaging (DTI), which can identify broken connections between nerve cells and magnetic resonance spectroscopy (MRS), which looks at chemical changes. Biochemical methods attempt to find changes (generally in blood or cerebral spinal fluid), which are correlated with trauma to the brain.

Different disciplines address different portions of the effect size spectrum from psychology, which deals with effect sizes smaller than *d* = 1.0 to engineering, which deals with effect sizes >5.0. While the term “effect size” is used in psychology, the concept is identical to the engineering “signal-to-noise ratio” sometimes encountered in physiological studies. The concepts are the same in that the difference between the means can be conceptualized as the true “signal” and the variance (or SD or error) as the “noise.”

There does not seem to be a comparable term in biochemistry where the less manageable terms “limit of detection” or sensitivity are used. This choice may be due to the fact that chemical tests are referenced to a 0 value rather than a contrasting population.

Radiology and medicine in general deal with signs and symptoms with such extreme effect size that they seem to have experienced no need for the concept. A broken bone, for instance, is so obvious that detection is not an issue. This is the realm of the “Augenblick” (blink of the eye) diagnosis.

## Two Meanings of “mTBI”

It is unfortunate, but true, that in describing a measurement method as an “mTBI diagnostic,” little distinction is made of the immediate effects of a blow to the head versus the chronic effects.

A blow to the head produces an immediate nerve block in the neurons of the brain. Trauma affects the ability of the axons to conduct action potentials and results in an immediate state of unconsciousness sometimes accompanied by motor disturbance. With severe trauma, this transitions into coma and permanent disability. With less severe trauma, the patient recovers consciousness in a few minutes followed by a period of gradual recovery of function. The period of immediate severe cognitive impairment is readily observable and presents few diagnostic problems.

By contrast, the syndrome of mild cognitive impairment following a blow to the head is much more difficult to appreciate behaviorally and to measure using assessment instruments. This entity has been termed as “mild TBI” (mTBI). The source of the impairment presumably is a loss of a proportion of the nerve connections that were damaged in the initial insult.

Measurement of severe mental impairment in dementia has long been done to using “mental status examinations” such as the Folstein mini-mental state exam [MMSE; (5)]. The military has used the MACE, the military acute assessment evaluation, an examination that closely resembles the MMSE. While mental status examination is useful where cognitive deficits are severe, such examinations lack the power to distinguish mild cognitive deficits.

The ability of the MACE to distinguish impaired versus unimpaired cognitive ability drops off rapidly. On the day of injury McCrea’s work (6) suggests a *d* of 1.17 in a sports concussion setting. Kennedy’s study (7) of soldiers examined <6 h after injury suggests a *d* of 1.12; by contrast, those examined between 6 and 12 h after injury had *d* of 0.53 (8). Coldren et al. (9) looked at soldiers in combat operations more than 12 h after injury and found a *d* of 0.31. These findings are consistent with a dramatic decline in *d* over the space of hours. Translating this into effectiveness of diagnosis, a drop from 80% correct diagnosis to 59%.

From the perspective of measurement theory, cognitive impairment in the acute state is so great that the difference between the means of the impaired and unimpaired populations is so great that it overwhelms the large variance in the behavioral measurement instruments and produces a large effect size. Impairment in the chronic, mTBI state, on the other hand, is too small to be measured effectively with short mental status exams.

It should be noted that inability of a test distinguish a deficit is not the same as saying no deficit exists. The existence of mild TBI is supported by numerous examples of anecdotal evidence.

Mild traumatic brain injury varies as to both severity and time since injury. During the acute period of victims experience, an initial mental impairment that subsides to a point after which it is unrecognizable to observers and unmeasurable by standard mental status examination. Those injured may continue to experience subjective impairment, but objective measurement of impairment is needed for making decisions concerning battlefield triage, treatment, and return to duty. The lack of objective measurement hinders the gathering of epidemiological data and successful therapeutic development efforts.

## Sample Size

The relationship between effect size and sample size is interesting. A common use of effect size is in determining the number of subjects to use in a study in order to be satisfied that a difference, if present, will be detected and that a difference, if detected, is real. Power analysis is the technique that is used for establishing the number of subjects that will be required given a known or estimated effect size. To distinguish small effect sizes large numbers of subjects are recommended. Large effect sizes, on the other hand, are easily discriminated. The relationship between effect size and the number of subjects needed is illustrated below (Figure 1B).

## Improving the Diagnostic

One of the advantages of conceptualizing diagnostics in terms of classical measurement theory is that it leads naturally to a rational method toward the goal of maximizing the effect size. Classical theory is based on the conception that the measured value has two additive components, a “true” value, plus or minus some “error” value. By repeatedly measuring, the mean and the SD can be estimated. The mean is the best estimate of the true value; the SD is the best estimate of the overall error.

If the difference between the two means is the true measure of the difference between the impaired and unimpaired groups, improvements in the measurement method will not substantially change that difference, hence not changed the effect size. Reducing the *error* in the measurement, however, will improve the ability of the measurement error to distinguish between the two populations.

Steps to improve the diagnostic can be easily quantified. Variances (squared SDs) can be added and subtracted. Components of the overall error can be identified by measuring them and assessing their overall contribution to the error using analysis of variance or regression techniques.

In the process of test development, the validation of the instrument is expensive and time consuming. But, since the effect size depends on both the variance of the unimpaired (normal or control) population, much developmental work can be done using readily available subjects without seeking out the more elusive concussed subjects. An axiom of classical measurement theory is that a test cannot be valid if it is not reliable. Much can be done to increase effect size by increasing the reliability of the test prior to engaging in validity studies. One source of unreliability that can be addressed early is inconsistency in administration of the instrument. Another way to increase reliability is to increase the number of measurements. Psychologists have long used the Spearman–Brown formula (10, 11) to estimate the increase in reliability that can be gained by adding items to a test battery. The same logic can be applied to other measurement methods. Some physiological instruments such as eye tracking devices and electroencephalographic techniques lend themselves readily to acquiring multiple measurements.

Because variance (but not SDs) can be added and subtracted, in the situation in which the unimpaired population (A) is less heterogeneous than the impaired population (B) the variance of B minus A gives the variance due to impairment. This variance, as noted above, may be due to differences in time elapsed since exposure and/or different levels of severity. One way to increase effect size then would be to select subjects for the impaired population who were more identical in these features. Errors in subject selection can definitely increase variance and reduce effect size by blurring the distinction between controls and affected individuals. Impaired individuals, perhaps from head trauma long ago, if included in the control group increases variance in one of the groups being compared. An individual who may have had a head injury, but were unaffected, if included in the impaired group, affect variance on the other side. Clearly, for extreme care must be exercised in subject selection. A good strategy might be to start with extreme examples and work toward subject groups more representative of the population as a whole. For a review addressing recruitment of narrowly defined populations, see Sadler et al. (12).

## Discussion and Summary

The development of effective diagnostics for mTBI need not have, as a pre-requisite, a rationally developed definition. Instead, an empirically developed “operational” definition should be the aim. For example, in practice, clinicians do not refer to an abstract definition of diabetes, but rather use test results, blood glucose, A1C levels as the operationalized definition of the disorder.

The practical meaning of small and large effect sizes has been described. Some landmarks along the spectrum of effect size are provided. Different researchers who are involved with mTBI research address different portions of that continuum: epidemiologists, psychologists and psychiatrists, physiologists, biochemists, physicians (including radiologists), and engineers. Different specialties may use different terms for the concept of effect sixe. For example, there is an equivalence between the psychologist’s effect size and the engineer’s signal-to-noise ratio. One of the objects of this paper has been to furnish useful context for appreciating the meaning of effect size.

Two arenas are discussed in which and understanding of effect size can be useful: in comparing diagnostics on the same scale for choosing potential diagnostic approaches for funding and for developing the discriminability of diagnostics by a focus on their reliability (low variance). The effect size statistic allows comparison of the effectiveness of different diagnostic methods no matter how diverse or different they are. For the investigator trying to improve on a given method, the paramount need for reducing variance becomes obvious. Effect size, the central measure guiding diagnostic development, is a statistic that normalizes mean differences over variance. And, the utility of approaching diagnostic development from the standpoint of reducing variance was discussed.

Two very distinct phases follow a concussive event. These need to be considered separately in diagnosis: the immediate high-effect size, acute impairment following head trauma, and the low-effect size chronic after effects. The acute symptoms are obvious enough to be assessed using almost any instrument and resemble the decline in abilities measured by general mental status testing.

The effect size approach outlined can be used in all of the four main methods proposed for mTBI diagnosis. The concept of effect size is well established in psychology and, in another form, in engineering. In biochemical and radiological studies, it is perhaps not as widely used as it should be.

Several related issues have not been discussed in order to highlight the meaning of the effect size statistic (13). There is a long-standing debate over the extent to which chronic mTBI symptoms following combat head trauma overlap with symptoms caused by combat stress, the symptom cluster known as post-traumatic stress disorder. That debate is not addressed here. The weighting of the cost of the two kinds of mistakes, false negatives and false positives, is an important issue that begs attention, but it is independent of the concerns surrounding development of an objective diagnostic measurement instrument. Likewise, a concern about mTBI as a relatively rare disorder and the effect that has on the number of false positives has been omitted. Subtleties of the mathematics of the concept of effect size have been minimized here in order to emphasize the practical conceptual meaning of the statistic. The issue of extending the two-group comparison discussed here to multiple disorders and using multiple measurement methods has not been included.

An outline of a classical measurement approach to development of an empirical, operational diagnostic has been described. For such development, the essence of a good diagnostic is its ability to distinguish impaired from unimpaired individuals. The measure of discriminability is the statistic effect size.

## Conflict of Interest Statement

The author declares that the research was conducted in the absence of any commercial or financial relationships that could be construed as a potential conflict of interest.

## Disclaimer

The views and opinions expressed in this article are those of the author and do not necessarily reflect the official policy or position of any agency of the U.S. government.
